# Supplementary material for: Lasting effects of the COVID-19 pandemic on language processing
Source: PLoS One. 2022 Jun 15;17(6):e0269242. doi: 10.1371/journal.pone.0269242 (PMC9200165; doi:10.1371/journal.pone.0269242)
Supplement: S1 Text — (DOCX) [file pone.0269242.s001.docx]

**Lasting Effects of the COVID-19 Pandemic on Language Processing**

**S1 Text: Supplemental Method and Results**

This document includes supporting information for the accompanying manuscript, *Lasting Effects of the COVID-19 Pandemic on Language Processing*. A table of contents for this document follows:

| Method: Deviations from Preregistrations | p. 2 |
| --- | --- |
| Method: Data Exclusion | p. 2 |
| Participant exclusion criteria | p. 3 |
| Item exclusion criteria | p. 3 |
| Table A | p. 4 |
| Changes in recruitment and criterion-setting to improve data retention | p. 4 |
| Effects of experimental design on exclusions | p. 5 |
| Method: Model-Fitting Strategy | p. 6 |
| Results: Preregistered Analyses | p. 6 |
| Cross-experiment analysis for preregistered experiments only | p. 6 |
| Analyses that included acceptable alternative responses | p. 7 |
| Table B | p. 8 |
| Results: Exploratory Analyses | p. 9 |
| Awareness | p. 9 |
| Self-priming | p. 10 |
| Supplementary References | p. 10 |

**Method: Deviations from Preregistrations**

All details regarding data collection, trial exclusions, analytic techniques, and hypothesis testing methods in Experiments 1b, 2a and 2b were identical to what was described in their respective preregistrations except for two discrepancies in sample sizes. In Experiment 1b, we declared that we had collected data from 240 participants; due to a technical error, we had actually collected data from 237 participants (99% of the planned *N*). In Experiment 2a, we planned to collect data from 480 participants, with additional data collected as necessary to ensure a minimum *n* of 120 for each of four experimental lists; due to a technical error, we ultimately collected data from a total of 469 participants (98% of the planned *N*), with a minimum *n* of 113 for each list. As these deviations were not noticed until after data analysis and they were relatively minor, we did not correct them.

**Method: Data Exclusion**

As noted in the manuscript, our experimental design assumed that participants were paying attention, were motivated to respond appropriately, and were able to hear the stimuli clearly. Accordingly, we discarded data from participants who did not produce enough responses that were consistent with the auditory input (e.g., producing a word like *mask*, *task*, *ask* or *flask* to the stimulus *#ask*). Similarly, our experimental design assumed that each sound token could be clearly heard by participants, who could plausibly respond with the target or competitor words. Accordingly, we discarded data from stimulus quadruplets (both COVID and CONTROL pairs) for which one or both word pairs did not elicit enough responses that matched either the target or competitor words (pooling across COUGH and NOISE conditions). Importantly, if a COVID word pair failed to meet inclusion criteria and was excluded, its CONTROL word pair was excluded as well (and vice-versa), thereby maintaining the use of CONTROL pairs as a baseline against which to measure COVID-related effects. Table A shows the total number of included participants, item quadruplets, and trials for each experiment, as well as the extent to which exclusion criteria were preregistered.

**Participant exclusion criteria.** The minimum number of input-consistent responses that a participant had to produce to be included was preregistered (except where noted) and varied by experiment: *Exp 1a*: 0% (no participant exclusions; this experiment was not preregistered); *Exp 1b*: 37.5%; *Exp 2a*: 40%; *Exp 2b*: 35%. These criteria led to participant inclusion rates of 96-100% for Experiments 1a, 1b and 2b; and 44% for Experiment 2a.

**Item exclusion criteria.** The minimum number of matching responses that a stimulus quadruplet had to elicit (separately for both COVID and CONTROL pairs) to be included was preregistered (except where noted) and varied by experiment: *Exp 1a*: 10% (this experiment was not preregistered); *Exp 1b*: 5%; *Exp 2a*: 20%; *Exp 2b*: 10%. These criteria led to stimulus quadruplet inclusion rates of 70-94% for Experiments 1a, 1b and 2b; and 30% for Experiment 2a. (Note that the stimuli in Experiments 2a and 2b were identical; however, participant data quality for Experiment 2a was low enough that stimuli failed to reach criteria.)

**Table A.** Rates of data inclusion for participants, item quadruplets, and trials.

| Experiment(s) | Criteria Preregistered | Participants | | Item Quadruplets | | Trials | |
| --- | --- | --- | --- | --- | --- | --- | --- |
| 1a | No | 229/237 | 96.6% | 13/16 | 81.2% | 1604/3792 | 42.3% |
| 1b | Pre-analysis | 232/237 | 97.9% | 15/16 | 93.8% | 1827/3792 | 48.2% |
| 2a | Pre-collection | 206/469 | 43.9% | 6/20 | 30.0% | 883/9360 | 9.4% |
| 2b | Pre-analysis | 232/240 | 96.7% | 14/20 | 70.0% | 1873/4800 | 39.0% |
| 1a, 1b, 2a, 2b |  | 899/1183 | 76.0% | 27/33 | 81.8% | 6187/21744 | 28.5% |
| 1a, 1b, 2b |  | 693/714 | 97.1% | 27/33 | 81.8% | 5304/12384 | 42.8% |

*Note*. The Criteria Preregistered column indicates whether the data exclusion criteria for each experiment were pre-registered, and (if so) whether they were pre-registered prior to data collection or prior to unblinded data analysis (for details, see the *Changes in recruitment and criterion-setting to improve data retention* section below). Fractions and accompanying percentages in the Participants and Item Quadruplets columns represent the number of participants (or item quadruplets) that contributed one or more trials to analyses divided by the total number of participants who completed the experiment (or item quadruplets that were presented to participants). Fractions and accompanying percentages in the Trials columns represent the proportion of trials used in analyses. Due to the combination of many participants and very low data inclusion rates for Experiment 2a, cross-experiment inclusion rates are shown with and without that experiment.

**Changes in recruitment and criterion-setting to improve data retention.** The low rate of data usability for Experiment 2a, which was the first preregistered experiment conducted as part of this study, led us to make two changes for subsequent experiments (Exps. 1b and 2b). First, we changed our method of online participant recruitment to ensure better data quality by adding greater screening restrictions based on eligible participants’ study completion history, as described in the *Participants* section of the manuscript. Second, we approached criterion-setting differently. As described in the preregistrations, we determined the exclusion criteria for Experiments 1b and 2b after data were collected but before they had been analyzed in ways that were relevant to our hypotheses. Specifically, the trial-level responses were coded, after which a condition-blinded version of the data file was used by one experimenter to determine how different exclusion criteria would affect the total amount of usable data for analysis. After a final set of criteria were selected to balance data quality and data retention, the preregistration was submitted; only then were the data unblinded and analyzed. Together, these changes led to better rates of data inclusion for subsequent experiments.

**Effects of experimental design on exclusions.** As noted in the manuscript, 62.1% of responses did not match either the TARGET or the COMPETITOR; this is what drove item exclusions. The high exclusion rate of non-matching responses reflects three constraints imposed by our experimental design. First, our choice of stimuli was heavily curtailed by both semantic and lexical factors, as we first needed a COVID-TARGET word which was strongly pandemic-related; then another word (COVID-COMPETITOR) which differed by exactly one sound and had no plausible pandemic relationship; and, finally, a second pair of words (CONTROL-TARGET and CONTROL-COMPETITOR) which differed from each other by the same sound at the same word location (initial, medial, or final) as the COVID pair, were both pandemic-unrelated, and were (collectively) matched with COVID-TARGET stimuli on pre-pandemic lexical frequency. These constraints greatly reduced the space of possible stimuli, and thus there was a tradeoff between maximizing the number of stimulus quadruplets vs. stimulus quality. (For example, multi-syllabic words have a higher rate of restoration (1), but many of our stimuli were monosyllabic.) Second, these same constraints meant that many stimuli came from dense phonological neighborhoods, leading to multiple input-consistent competitors (e.g., *ask*, *flask*, *bask*) that participants might reasonably perceive which were neither the TARGET nor the COMPETITOR. We attempted to minimize the size of the phonological neighborhood around the minimal pair phoneme which would ultimately be blocked out by the noise, though this was not always possible. However, including input-consistent competitors in the analyses did not change the statistical significance of any results in the cross-experiment analysis (for details, see *Analyses that included acceptable alternative responses*). Finally, participants completed the experiment using their own listening equipment in their own homes, as opposed to high-quality audio equipment in a monitored, noise-shielded laboratory setting, as is typically the case for such experiments. Thus, it is likely that there was high variability in the listening experience, background noise, and sound fidelity between participants, to a greater degree than for prior phonemic restoration experiments.

**Method: Model-Fitting Strategy**

For all mixed-effects models, we followed a three-step strategy to facilitate convergence. First, we fit a model with a maximal random effects structure: random intercepts for participants and item quadruplets, all within-factor random slopes and their interactions, and correlations between random slopes. If this model did not converge, we removed correlations between random slopes. If the resulting model still did not converge, we identified random slopes accounting for less than 1% of the variance of their associated random factors, then removed all such slopes simultaneously (2). This always resulted in convergence.

**Results: Preregistered Analyses**

**Cross-experiment analysis for preregistered experiments only.** Experiment 1a was not preregistered. Combining experiments in a single analysis can yield spurious results if even some of the experiments were analyzed using questionable research practices. Although we did not employ such practices here, we erred on the side of caution by repeating the cross-experiment analysis using only data from experiments that had preregistered analysis plans (Experiments 1b, 2a, 2b).

Results showed an identical pattern of statistical significance to those obtained when Experiment 1a was included. Participants heard ambiguous input as COVID-TARGET words more often than CONTROL-TARGET words, *B* = 1.55, 95% CI = [0.74, 2.37], *z* = 3.13, *p* = .001. They restored ambiguous input to TARGET words more often in the presence of an interfering COUGH compared to NOISE for COVID word pairs, *B* = 2.67, 95% CI = [0.21, 0.89], *z* = 2.67, *p* = .004, but not for CONTROL word pairs, *B* = 0.00, 95% CI = [-0.41, 0.40], *z* = 0.00, *p* = .501, yielding a significant interaction, *B* = 0.55, 95% CI = [0.12, 0.98], *z* = 2.10, *p* = .018.

**Analyses that included acceptable alternative responses.** The analyses reported in the manuscript restricted the set of analyzed responses to the 37.9% that matched either the TARGET (e.g., *mask*) or the COMPETITOR (*task*). An additional 16.0% of responses were consistent with the auditory input, comprising words like *ask* and *flask*. We excluded these input-consistent competitor responses from our primary set of analyses because properties of the TARGET and COMPETITOR words had been controlled but their phonological neighborhoods were not. However, as specified in each experiment’s preregistration, we conducted a secondary set of analyses in which these responses were included and coded as non-target responses (0). The results of these analyses are shown in Table B for each experiment individually, all experiments together, and all preregistered experiments together. Importantly, the pattern of statistical significance in the cross-experiment analyses was identical to that obtained when input-consistent competitor responses were excluded.

**Table B.** Results of all hypothesis tests conducted for individual experiments and across experiments when input-consistent competitor responses were included.

| Question | Comparison | Experiment | *B* | 95% CI | *z* | *p* |
| --- | --- | --- | --- | --- | --- | --- |
| Did the pandemic increase COVID-related responses? | COVID/Noise - Control/Noise | 1a | 1.41 | [ 0.25, 2.58] | 1.99 | **.023** |
|  |  | 1b | 1.61 | [ 0.52, 2.69] | 2.44 | **.007** |
|  |  | 2a | 2.68 | [ 1.44, 3.92] | 3.56 | **< .001** |
|  |  | 2b | 1.85 | [ 0.98, 2.72] | 3.50 | **< .001** |
|  |  | All | 1.71 | [ 0.97, 2.45] | 3.80 | **< .001** |
|  |  | Preregistered | 1.99 | [ 1.21, 2.77] | 4.21 | **< .001** |
| Do coughs increase COVID-related response? | COVID/Cough - COVID/Noise | 1a | 0.65 | [ 0.26, 1.05] | 2.70 | **.003** |
|  |  | 1b | 0.34 | [-0.25, 0.94] | 0.95 | .172 |
|  |  | 2a | 0.68 | [ 0.03, 1.33] | 1.73 | **.042** |
|  |  | 2b | 0.53 | [ 0.14, 0.92] | 2.25 | **.012** |
|  |  | All | 0.54 | [ 0.25, 0.82] | 3.11 | **< .001** |
|  |  | Preregistered | 0.36 | [ 0.08, 0.65] | 2.08 | **.019** |
| Do coughs increase COVID-unrelated responses? | Control/Cough - Control/Noise | 1a | 0.22 | [-0.23, 0.67] | 0.79 | .215 |
|  |  | 1b | 0.01 | [-0.64, 0.66] | 0.03 | .490 |
|  |  | 2a | 0.26 | [-0.68, 1.19] | 0.45 | .327 |
|  |  | 2b | -0.19 | [-0.68, 0.29] | -0.65 | .744 |
|  |  | All | 0.04 | [-0.30, 0.37] | 0.18 | .430 |
|  |  | Preregistered | -0.02 | [-0.35, 0.32] | -0.07 | .530 |
| Do coughs increase COVID-related responses more than COVID-unrelated responses? | (COVID/Cough - COVID/Noise) - (Control/Cough - Control/Noise) | 1a | 0.44 | [-0.03, 0.91] | 1.54 | *.062* |
|  |  | 1b | 0.33 | [-0.14, 0.80] | 1.16 | .122 |
|  |  | 2a | 0.43 | [-0.48, 1.33] | 0.78 | .218 |
|  |  | 2b | 0.73 | [ 0.21, 1.24] | 2.32 | **.010** |
|  |  | All | 0.50 | [ 0.17, 0.83] | 2.49 | **.006** |
|  |  | Preregistered | 0.38 | [ 0.06, 0.69] | 1.96 | **.025** |

*Note*. Effect slopes (*B*) represent the change in log-odds ratios between conditions. All predictors were coded so as to yield a numerically positive effect if the answer to the corresponding question was affirmative. As specified in preregistrations, all hypothesis tests were one-tailed. *p*-values are shown in **bold** for statistically significant effects (*p* < .05) and in *italics* for marginally significant effects (.05 < *p* < .10). Rows in which Experiment is equal to Preregistered reflect analyses of data from preregistered experiments only (1b, 2a, 2b).

**Results: Exploratory Analyses**

**Awareness.** As described in the manuscript, we considered whether our results were driven by awareness of the purpose of the study rather than unconscious reorganization of the lexicon. After Experiments 1b and 2b, participants completed a post-experiment questionnaire in which they answered the question “How likely do you think it is that the experiment was about each of the following?” by indicating their response on a sliding scale from 1 (not likely) to 5 (very likely) for each of 11 topics, one of which was “Understanding words related to covid-19” (see https://osf.io/dxc97/ for the complete questionnaire). For the purposes of determining awareness, we erred on the side of caution by treating participants as aware if they responded to this item with a score of 3 or greater (*n*=178 participants with usable data) and as unaware if they responded with a score of 1 or 2 (*n*=290 participants with usable data). Then, we repeated the analyses and contrasts separately for each group.

The pattern of statistical significance for each group matched that of the analysis with all participants regardless of awareness. Participants in both groups heard ambiguous input as COVID-TARGET words more often than CONTROL-TARGET words [Unaware: *β* = 1.39, 95% CI = [0.63, 2.16], *z* = 2.99, *p* = .001; Aware: *β* = 1.87, 95% CI = [1.15, 2.60], *z* = 4.25, *p* < .001]. They restored ambiguous input to TARGET words more often in the presence of an interfering COUGH compared to NOISE for COVID word pairs [Unaware: *β* = 0.50, 95% CI = [0.09, 0.90], *z* = 2.03, *p* = .021; Aware: *β* = 0.56, 95% CI = [0.18, 0.95], *z* = 2.42, *p* = .008], but not for CONTROL word pairs [Unaware: *β* = -0.17, 95% CI = [-0.68, 0.35], *z* = -0.54, *p* = .704; Aware: *β* = -0.21, 95% CI = [-0.76, 0.33], *z* = -0.64, *p* = .739], yielding a significant interaction [Unaware: *β* = 0.66, 95% CI = [0.11, 1.21], *z* = 1.98, *p* = .024; Aware: *β* = 0.78, 95% CI = [0.19, 1.37], *z* = 2.17, *p* = .015].

**Self-priming.** As described in the manuscript, we considered whether our results could be attributable to semantic self-priming, in which restoring one COVID-related word (e.g., *mask*) would have increased the accessibility of other COVID-related words on subsequent trials. Such priming would have asymmetrically boosted target restoration in the COVID condition (*mask*, *sick*) because target words in the CONTROL condition (*map*, *kick*) were not selected to be semantically related. As this account would predict that target restoration rates should increase throughout the experiment due to priming, we repeated the cross-experiment analysis with an additional predictor – trial number, centered and scaled by the number of trials in each experiment – that was allowed to interact with all other fixed and random effects. Contrasts revealed that the effect of trial number was not statistically significant for COVID word pairs, either across noise types, *β* = -0.05, 95% CI = [-0.42, 0.31], *z* = -0.30, *p* = .767, or separately for either noise type [NOISE: *β* = -0.18, 95% CI = [-0.67, 0.31], *z* = -0.73, *p* = .466; COUGH: *β* = 0.07, 95% CI = [-0.41, 0.56], *z* = 0.29, *p* = .770], providing no evidence for a self-priming account.

**Supplementary References**

1. Samuel AG. Phonemic restoration: Insights from a new methodology. Journal of Experimental Psychology: General. 1981;110(4):474–94.

2. Bates D, Kliegl R, Vasishth S, Baayen H. Parsimonious mixed models [preprint]. 2018. Available from: https://arxiv.org/abs/1506.04967
