# Supplementary material for: Lasting effects of the COVID-19 pandemic on language processing
Source: PLoS One. 2022 Jun 15;17(6):e0269242. doi: 10.1371/journal.pone.0269242 (PMC9200165; doi:10.1371/journal.pone.0269242)
Supplement: Attachment — Submitted filename: Gloves_Response to Reviewers_R1.docx [file pone.0269242.s002.docx]

*Next, some thoughts from a phonetician: fricatives and voiceless stops have real noise components, and I would have expected that this would make listeners more likely to restore towards "task" than towards "mask." I'm not intimately familiar enough with the phoneme restoration literature to know whether this effect has been documented or not. Either way, this is of course not a confound for your effect in cases like mask/task, quite the opposite, and strengthens the case that a phonetic phenomenon is not responsible for the higher proportion of COVID target restorations. Just something to think about and comment on, if you wanted to.*

We added a comment that our design obviates any concerns about different phonemic/phonetic properties between different types of consonants, as they are matched in the COVID and CONTROL conditions (pg. 9-10):

“…this ensured that any differences between phonemes that might affect restoration (e.g., the presence of noise components in fricatives and voiceless stops) would affect the COVID and CONTROL conditions equally.”

*I appreciate that all the data and materials are available at the additional link, but I think it would be nice to have a clearer explanation of how experiments 1 and 2 differed from each other in the text, and also to have a stimuli table.*

We have added a comment about the differences between Exps 1 and 2 on page 9:

“The only experimental design difference between Experiments 1 and 2 was the specific stimuli used.”

We have also added a stimuli table (Table 1, page 12).

*I'd like to know how many minutes the study typically lasted if participants were only paid $2.50. It's important to be transparent about how we treat participants.*

We have added a note about the length of the study (10 minutes) on page 7.
